# Spatial variation in risk and consequence of *Batrachochytrium salamandrivorans* introduction in the USA

**DOI:** 10.1098/rsos.150616

**Published:** 2016-02-17

**Authors:** Katherine L. D. Richgels, Robin E. Russell, Michael J. Adams, C. LeAnn White, Evan H. Campbell Grant

**Affiliations:** 1 US Geological Survey National Wildlife Health Center, 6006 Schroeder Road, Madison, WI 53711, USA; 2 Department of Pathobiological Sciences, School of Veterinary Medicine, University of Wisconsin—Madison, 2015 Linden Drive, Madison, WI 53706, USA; 3 US Geological Survey Forest and Rangeland Ecosystem Science Center, 3200 SW Jefferson Way, Corvallis, OR 97331, USA; 4 US Geological Survey Patuxent Wildlife Research Center, SO Conte Anadromous Fish Research Laboratory, 1 Migratory Way, Turner Falls, MA 01376, USA

**Keywords:** fungal pathogen, amphibians, urodeles, Caudata, invasive species, disease

## Abstract

A newly identified fungal pathogen, *Batrachochytrium salamandrivorans*(*Bsal*), is responsible for mass mortality events and severe population declines in European salamanders. The eastern USA has the highest diversity of salamanders in the world and the introduction of this pathogen is likely to be devastating. Although data are inevitably limited for new pathogens, disease-risk assessments use best available data to inform management decisions. Using characteristics of *Bsal*ecology, spatial data on imports and pet trade establishments, and salamander species diversity, we identify high-risk areas with both a high likelihood of introduction and severe consequences for local salamanders. We predict that the Pacific coast, southern Appalachian Mountains and mid-Atlantic regions will have the highest relative risk from *Bsal*. Management of invasive pathogens becomes difficult once they are established in wildlife populations; therefore, import restrictions to limit pathogen introduction and early detection through surveillance of high-risk areas are priorities for preventing the next crisis for North American salamanders.

## 1. Background

*Batrachochytrium salamandrivorans* (*Bsal*), a recently described chytrid pathogen, causes morbidity and mortality in salamanders [1,2] and is expected to pose a substantial risk to global amphibian biodiversity. *Bsal* was first described from declining fire salamander (*Salamandra salamandra*) populations in Belgium and The Netherlands [1–3] and is suspected to have been introduced into Europe via the importation of Asian salamanders (*Cynops*spp. and *Paramesotriton* spp. [2]) for the pet trade. Indeed, *Bsal* was subsequently identified on commercially traded salamanders in the UK and Germany [4,5]. Concern for the spread of *Bsal* is compounded by the continued spread of a related chytrid pathogen, *B. dendrobatidis* (*Bd*), which has contributed to global population declines (more than 200 species affected [6]) and the classification of amphibians as the most threatened vertebrate taxa worldwide [7]. Similar to the generalist nature and high pathogenicity of the pandemic strain of *Bd*[6], *Bsal* caused substantial mortality in over half of salamander species tested in laboratory infection studies (USA: 2/7 species tested; globally: 12/23 species tested [2]). Although preliminary testing of archived salamander specimens and native salamander populations has failed to detect *Bsal* in the USA, only a fraction of North American species have been tested (185 individuals from 7 of 191 species [8,9]). Based on the phylogenetic relationship with *Bd*, wide host range, and high pathogenicity [2], *Bsal* introduction into the USA could threaten North American salamander biodiversity.

Predicting the potential distribution of a pathogen in novel habitats is an integral part of a formal disease-risk analysis that evaluates the consequences of an invasive disease. Two modelling approaches are commonly used to predict shifts in species distributions: (i) correlative approaches that use statistical relationships between environmental characteristics and observed occurrences and (ii) mechanistic approaches that use relationships between environmental characteristics and organismal performance that are independent of current distributions [10]. Recently, a correlative approach was used to estimate the invasive range of *Bsal* for North America [11]. This species distribution model characterized the realized niche of potential reservoir species in their native range in Asia (identified from archived wild caught specimens [2,11]), and identified similar environmental conditions in the USA. This approach identified only climatic conditions amenable to the known host species and did not consider the ecology of the pathogen itself. Correlative species distribution models that are based on the realized niche of invasive species in their native range can underestimate the resulting invasive range [12]. Mechanistic species distribution models, on the other hand, rely on the underlying mechanisms contributing to a pathogen’s range [1,13] and may provide better predictions of range shifts during invasions [10]. More importantly, presenting multiple approaches to estimate potential distribution of pathogen invasion will increase overall confidence in predicted areas of agreement and highlight areas of disagreement, where the effect of underlying assumptions on risk predictions can be tested should introduction occur.

Emerging infectious diseases necessitate that decision-makers make decisions in the face of uncertainty. This is particularly true for wildlife diseases because pathogens established in wild populations are extremely difficult to eradicate [14]. Providing decision-makers with tools, such as multiple risk assessments based on varying underlying assumptions (about the host or pathogen for example), allows them to weigh the strength of particular predictions when making decisions about management or surveillance for a pathogen, and to update their confidence in underlying assumptions as more information becomes available. Here, we present an alternative risk assessment that uses a mechanistic approach to predict suitable habitat for *Bsal*, and combine this with a formal assessment of the risk of *Bsal* introduction to US salamander populations. Specifically, we followed the World Organisation for Animal Health disease-risk assessment framework [15], where the total risk to native amphibian populations from *Bsal*in the USA is the combination of two events: (i) where introductions are likely to occur (i.e. introduction assessment) and (ii) if introduced, where suitable habitat and high species diversity overlap (i.e. consequences assessment). We identify areas of the USA projected to be at the highest relative risk for *Bsal* introduction, and areas where consequences are expected to be most severe. We also discuss the current uncertainties and research priorities needed to better understand and manage the threat to US amphibians from *Bsal*.

## 2. Methods

We used a hierarchical multi-criteria evaluation approach [16], similar to those used by the International Union for Conservation of Nature’s red list and NatureServe to estimate risk of species declines when there is large uncertainty [17,18], and recently applied to predicting the risk of the fungus *Phytophthora ramorum* (the causative agent of sudden oak death) invasion in Oregon [19]. We combined factors with weighted linear combination (WLC), because it allows for measures of uncertainty (i.e. partial fuzzy set memberships [16]) and trade-offs among risk factors. We chose this combination method because, currently, how factors interact and affect overall risk from *Bsal* is unknown (i.e. the rank-importance and factor weights are equal for factors in each hierarchical level). Non-normally distributed factors were log transformed and all factors were linearly scaled from 1 to 4 (the lowest to the highest relative contribution to total risk; similar to [17]; electronic supplementary material, table S1). One of our objectives was to describe spatial variation in relative risk across the USA; therefore, the value of each contributing factor was calculated for each US county in the contiguous 48 states using ArcGIS v. 10.1 and Python v. 2.7 (raw and scaled values in the electronic supplementary material, table S1).

### 2.1 Introduction assessment

International trade has been implicated as the primary factor in the global spread of *Bsal* [2,4,5]. We assumed a linear relationship between proximity to areas with a high volume of amphibian trade (both international shipments through imports and domestic activity through pet stores) and the potential for *Bsal* introduction. While this is a simplification of the invasion process, it is the only step of the process that currently has available data. Thus, we estimated the relative potential of a *Bsal* introduction event from two derived factors, imports and the pet trade, representing the relative quantity of trade in live amphibians combined with initial spread from potential introduction locations. The import factor was based on records of all live salamander imports from 1 January 2010 to 31 December 2014 through a Freedom of Information Act request from the US Fish and Wildlife Service Law Enforcement Management Information System (LEMIS), which compiles data on international trade passing through all ports of entry. The LEMIS import information does not include the location of final sales of live salamanders; therefore, to estimate the potential introductions from the pet trade we used the US Census Bureau’s County Business Patterns [20] number of establishments reported as pet or pet supply stores (NAICS 45391) as a proxy for commercial trade of salamanders within the USA. The number of establishments and gross sales from the US Census Survey of Businesses [21] were highly correlated (*R*^2^=0.84, *p*<0.001) but sales for approximately half of all counties were not available due to business privacy rules.

**Figure 1.**
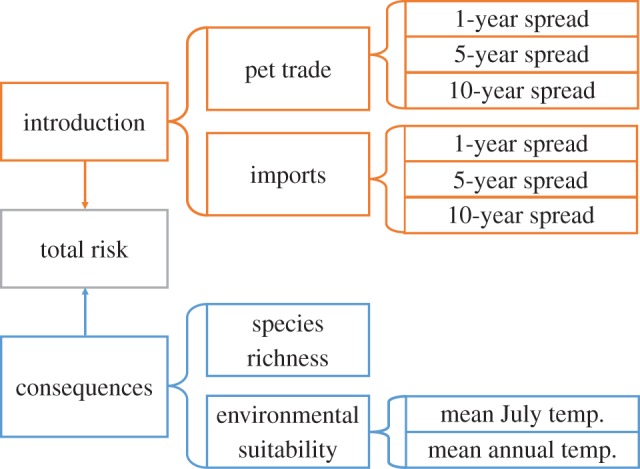
Conceptual diagram showing how each variable contributed to the estimated by-county introduction and consequences assessment for *Bsal*. Component variables were averaged and scaled for each hierarchical factor.

Even with highly rigorous surveillance efforts, the probability of detecting a single introduction event of a pathogen is low (e.g. [22]), so some spread from the initial importation location is expected before detection occurs. In other words, a particular county’s likelihood of introduction is a combination of both the number of pet stores and imports within that county and the number of pet stores and imports in neighbouring counties. To account for this combined risk, we first geolocated ports by their physical address and assigned each port the respective number of salamander imports from 2010 to 2014. Similarly, we assigned the number of pet establishments by county to the respective county centroid. We created three buffers of the port and pet establishment point layers to represent the potential spread of *Bsal* (based on the estimated spread for *Bd*, 26 km yr^−1^ [23]) 1, 5 and 10 years post introduction (26, 130 and 260 km), assigning each buffer the value of the buffered point. For each spread scenario, we summed the value of buffers that overlapped each county (‘intersect’, ArcGIS v. 10.1), creating six layers representing the three spread scenarios for ports and pet establishments. The three spread scenarios were then averaged to create an imports layer and a pet trade layer, where the potential for introduction declines as a function of distance from each type of introduction (figure 1; electronic supplementary material, table S1 and figure S1). The relative potential for introduction of *Bsal* was then determined by averaging the imports and pet trade layers.

### 2.2 Consequences assessment

The consequences of *Bsal*introduction were determined by two factors: environmental suitability and species richness (figure 1). Although many environmental requirements for *Bsal* survival and growth are unknown, physiological constraints (i.e. temperature of optimal growth and thermal maximum) for *Bsal* have been experimentally determined [1,13]. Mean annual air temperature and mean air temperature of the warmest month (July) were extracted by county from PRISM climate normals [24] using zonal statistics (spatial analyst toolbox in ArcGIS 10.1), which resulted in an average temperature value for all raster cells within each county. We scaled mean annual temperatures with the thermal optimum (15°C) as the highest score (4), representing ideal conditions for *Bsal* growth, and we scaled mean temperatures of the warmest month with greater than or equal to the thermal maximum of *Bsal* (25°C) as the lowest score (1), representing areas of the country that may have thermal refugia from *Bsal* infection. We then averaged these two temperature factors to derive environmental suitability (figure 1; electronic supplementary material, table S1).

Greater species richness may increase the number of available hosts, add highly susceptible species or add reservoir species, all of which may amplify disease transmission [25] (as seen for *Bd* in the USA [26]); therefore, species richness may be an important predictor of the consequences of *Bsal* introduction. To estimate species richness, we summarized the county-level occurrence of salamander species from a combination of NatureServe [27] and the USGS National Amphibian Atlas range maps [28]. We excluded two species from this list, *Plethodon ainsworthi* and the unisexual Jefferson salamander complex (*Ambystoma* sp.), because uncertainty exists in their classification as species [27]. We scaled species richness giving the county with the highest species richness the highest score (4); counties without salamander species were excluded from the risk assessment (*N*=116/3007, 4%; electronic supplementary material, tables S1 and S2). We calculated the potential for *Bsal*-caused declines (consequences assessment) by averaging environmental suitability and species richness. We averaged the introduction and consequences factors to estimate the total risk of *Bsal* by US county.

## 3. Results

**Figure 2.**
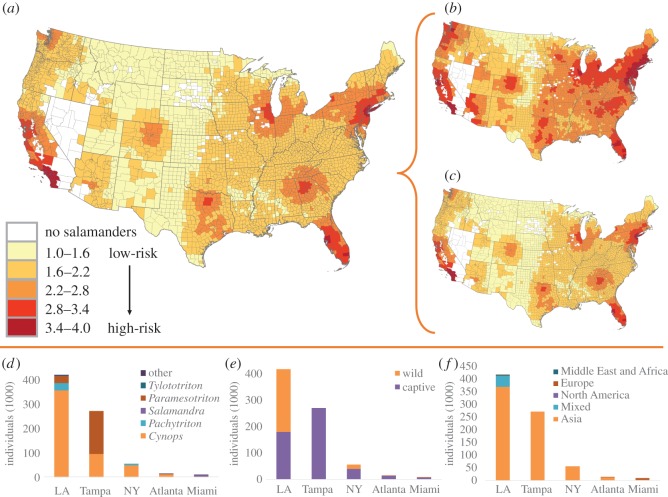
Heat maps of the USA showing the *Bsal* introduction assessment. The introduction assessment (*a*) is a combination of areas with high numbers of pet trade establishments (*b*) and high levels of imports (*c*). Salamander imports from the five most active ports (more than 99% of all imports) in 2010–2014 including by genera (*d*), by source (*e*) and by region of origin (*f*).

The USA imported at least 776 990 live salamanders from 1 January 2010 to 31 December 2014, through 14 ports of entry (155 398 yr^−1^±29 658 s.d.; electronic supplementary material, table S3). Seven ports contributed greater than 99% of all imports and had over 1000 total individuals imported: Los Angeles, CA (54%, 419 869), Tampa, FL (35%, 272 338), New York, NY (7%, 55 441), Atlanta, GA (2%, 13 310), Miami, FL (1%, 9370), San Francisco, CA (0.4%, 3243) and Chicago, IL (0.3%, 2178). Salamanders imported into the USA were predominantly reported to be captive bred (67%) and for commercial purposes (more than 99%). *Cynops* sp. (67%) and *Paramesotriton* sp. (27%), both suspected carriers of*Bsal*, were the two most common groups imported, followed by *Pachytriton* sp. (5%), *Salamandra salamandra* (1%; which accounted for the 1% of imports from Europe) and *Tylototriton* sp. (0.5%). Most shipments originated from Asia (93%), of which Hong Kong (70%) and China (27%) were the most commonly reported Asian origins (figure 2; electronic supplementary material, table S1 and figure S1).

There are 1235 US counties (40%) with pet or pet supply establishments according to the US Census Bureau’s County Business Patterns (2012). The average number of pet-related establishments per county was 7, with seven counties having at least 100 establishments: Los Angeles County, CA (283), Maricopa County, AZ (138), San Diego County, CA (134), Cook County, IL (133), Orange County, CA (106), King County, WA (104) and New York, NY (100). The resulting potential of *Bsal* introduction was highest near the major ports that also had numerous pet stores, which included central and southern Florida, southern California and near New York City, NY (figure 2; electronic supplementary material, table S1 and figure S1).

**Figure 3.**
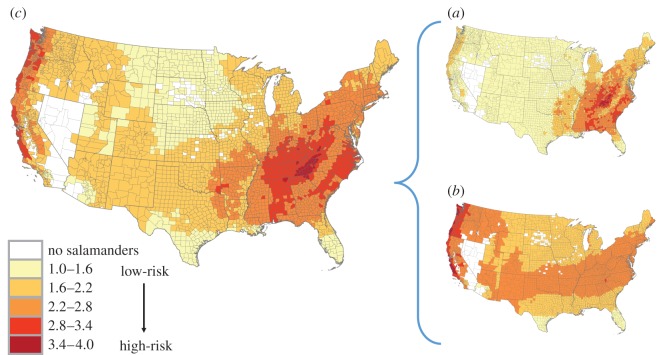
Heat maps of the USA showing the consequences of *Bsal* introduction. The consequence assessment (*c*) is a combination of species richness (*a*) and environmental suitability (*b*).

We identified the Pacific coast and Appalachian Mountains as the most likely—given an introduction—to have declines from *Bsal* based on environmental suitability and species richness. Environmental suitability indicated the southeast, southwest and Pacific regions of the USA as having mild climates well suited to *Bsal* growth (which are also suitable climates for salamanders, hence the concentration of species diversity). Species richness (range = 0–30) was the highest in the Appalachian Mountains and southeast USA (figure 3; electronic supplementary material, figure S2).

**Figure 4.**
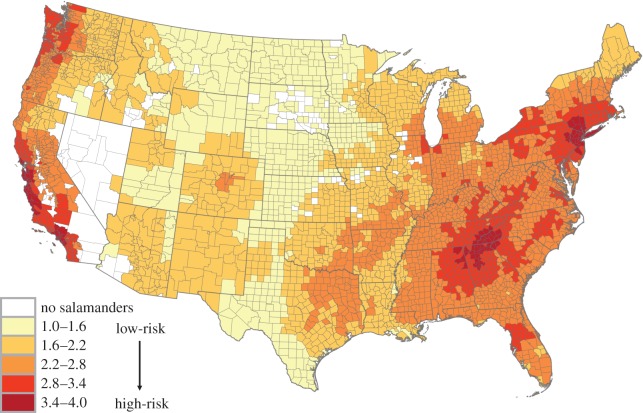
Heat map of the USA showing the total relative risk of *Bsal*to native US salamanders based on the introduction and consequences assessment.

The total risk of *Bsal* to US salamanders, based on the introduction and consequences assessment, is elevated throughout the eastern USA. Risk is expected to be the highest for the Pacific coast, southern Appalachian Mountains and mid-Atlantic regions (figure 4).

## 4. Discussion

Given the large number of suspected *Bsal* carriers imported into the USA each year (*Cynops* spp. and *Paramesotriton* spp., more than 100 000 yr^−1^), *Bsal* is likely to be introduced if no additional risk mitigation steps are taken. Though precise estimates for the invasion process (proportion of imported individuals infected, frequency of release of captive individuals, and contact of released animals with native amphibians) do not exist for *Bsal*, the establishment of invasive amphibians common in US amphibian trade (e.g. American bullfrogs, African clawed frogs, Asian newts [29–31]) and the patterns of global *Bd* spread [32] suggest these processes are also likely for *Bsal*. Currently,*Bsal* infection is known to be lethal to two US salamander species (of seven tested thus far [2]). While this number is small, these species are common and wide ranging (electronic supplementary material, figure S4). We also expect that as additional testing is completed, a larger fraction of US species may be found to be susceptible to *Bsal*. Thus, given the high likelihood that *Bsal* will be introduced and the potentially severe consequences, we categorize the risk of *Bsal* to US salamanders as high.

Our risk assessment predicted a higher total risk of *Bsal* for the northeastern and midwestern USA compared with Yap *et al*. [11], predicting a much larger area of the USA could be at risk for *Bsal* establishment. This is partially explained by the methodological differences used to estimate the potential range of *Bsal* in the USA between the two assessments. Our assessment uses the experimentally determined thermal range of *Bsal* rather than the native Asian host distribution (which may or may not be susceptible to infection across the entire range) used by Yap *et al*. [11]. Indeed, correlative species distribution models are suspected to under predict invasive species ranges [12], and mechanistic species distribution models often predict larger range shifts [10]. We also employed a more inclusive combinatorial method (WLC) compared with Yap *et al*. [11] (product); thus, our risk assessment is less restrictive and allows for trade-offs between factors. For example, an area with a high introduction score (1) but low consequences score (0) would have a medium risk score in our assessment ((1+0)/2=1/2) but a low-risk score in Yap *et al*.’s [11] assessment (1×0=0). Finally, we included an introduction assessment, which was not included in Yap *et al*. [11].

We assumed that *Bsal* has not been previously established in the USA; thus we estimated the potential for introduction as a function of volume of trade through ports and number of pet stores. However, the amount of salamander sales most probably varies among pet establishments due to local variation in the popularity of amphibians as pets and specialization of pet stores. Similarly, a proportion of salamander sales are through Internet breeders or hobbyist fairs, and the final location of these sales will be underrepresented in our analyses (as it is based on the physical location of the seller as reported on their tax forms, not the buyer). Additional introduction pathways are possible, such as from agricultural stowaways or infection in an already existing captive population. These represent a smaller and more difficult risk to assess, but are an important caveat—reducing commercial imports will not completely eliminate the risk of *Bsal*. If *Bsal* is established in the USA, additional information on interstate trade in salamanders, such as among-county movement of salamanders sold as fish bait, through Internet sales or hobbyists, would be useful to mitigate the impacts of human-mediated pathogen spread. The rate of spread of *Bsal* in wild populations is also uncertain and is expected to affect the estimate of spread from potential introduction locations. The reported rates of *Bd* spread have high variation (range from 1.1 to 282 km yr^−1^ [22]). In this assessment, we chose the estimate of *Bd* spread (26 km yr^−1^) that was associated with the most complete surveillance effort (the average rate of spread through Central America [22]). However, we acknowledge that the complexity of the terrain, typical movement of local amphibian communities and other factors may affect the realized invasion dynamics of *Bsal*.

When considering the impacts of an emerging wildlife disease, knowing which species may be susceptible, and thus experience population level declines, is critical. While laboratory exposure trials have shown that at least two US species, *Taricha torosa* and *Notophthalmus viridescens*, experience lethal responses to *Bsal* [2] (electronic supplementary material, figure S4), the responses of the other 189 US species to *Bsal* exposure is unknown. Similarly, we based the environmental suitability factor on the experimentally derived thermal range of *Bsal*, yet much is unknown about environmental characteristics in the field that will affect *Bsal* infection and transmission, such as amphibian behavioural thermoregulation and microhabitat use, and the persistence of the pathogen in various environmental conditions. For example, *Bd* was capable of infecting amphibians along a larger temperature profile than originally predicted using correlative environmental suitability approaches [32,33]. However, some of *Bd*’s wide range may be due to differential temperature profiles of diverse *Bd* strains [34,35]; an unexplored possibility for *Bsal*.

Control of wildlife diseases is more challenging and expensive than prevention [14] and expedient action is required to have the best opportunity to reduce the risk of *Bsal* invasion. There are few known viable treatment or management options for responding to the introduction of *Bsal* [36,37], and despite the fact that *Bd* has been identified and described for more than 15 years [38], there are currently no effective treatments available for wild populations. Strategies focused on prevention or reduction of introduction events remain the best control option for emerging diseases. Currently, *Bd* and ranaviruses are categorized as reportable diseases by the World Organisation for Animal Health and import restrictions and veterinary certifications can be required [15]. Similar import restrictions and health certificate requirements for *Bsal* would reduce the potential for introduction into the USA. Additional reductions in the potential for introduction are possible with the implementation of industry best-practices for treatment of waste, voluntary quarantine and treatment of imported amphibians (e.g. at least 10 days above 25°C [13]). Public awareness campaigns in partnership with private businesses and non-profit conservation organizations that highlight the potential for *Bsal* invasion and provide guidelines to prevent spread from captive populations may also be effective for reducing the risk of *Bsal* to wild populations.

## 5. Conclusion

Based on the information available for our assessment, the highest total risk of *Bsal*for wild salamander populations in the US occurs along the Pacific coast, southern Appalachian Mountains and mid-Atlantic regions. This risk assessment provides separate and combined estimates for two different types of risk, introduction and consequences, which can be used independently or together to inform policy decisions. Similarly, this study can be used to both assist in the development of a surveillance strategy for *Bsal* and direct applied research to areas with high uncertainty where additional information could make the largest impact on mitigation and prevention. Critical knowledge gaps which may refine future assessments include identifying the prevalence of *Bsal* in imported animals through sampling shipments upon arrival at US ports, determining the susceptibility of US salamander species through exposure experiments, and further refining the environmental conditions necessary for establishment of *Bsal*.

## Supplementary Material

Supplementary Tables - This file includes the raw and scaled values for the factors used in this risk assessment and other supplementary data tables.

## Supplementary Material

Supplementary Figures - This file includes additional figures for the Bsal risk assessment.

## Data accessibility

Datasets from this article are available in the electronic supplementary material.
