# Supplementary material for: Spatial variation in risk and consequence of Batrachochytrium salamandrivorans introduction in the USA
Source: R Soc Open Sci. 2016 Feb 17;3(2):150616. doi: 10.1098/rsos.150616 (PMC4785982; doi:10.1098/rsos.150616)
Supplement: Supplementary Figures - This file includes additional figures for the Bsal risk assessment. [file rsos150616supp2.pdf]

Figure S1: Heat maps of the US showing the components of the potential introduction of Bsal into the US. We combined hypothetical 1 yr (A,D), 5 yr (B,E), and 10 yr (C,F) spread of Bsal (based on the spread of Bd, Lips et al. 2008) to create the pet trade and imports variables (G,H respectively).

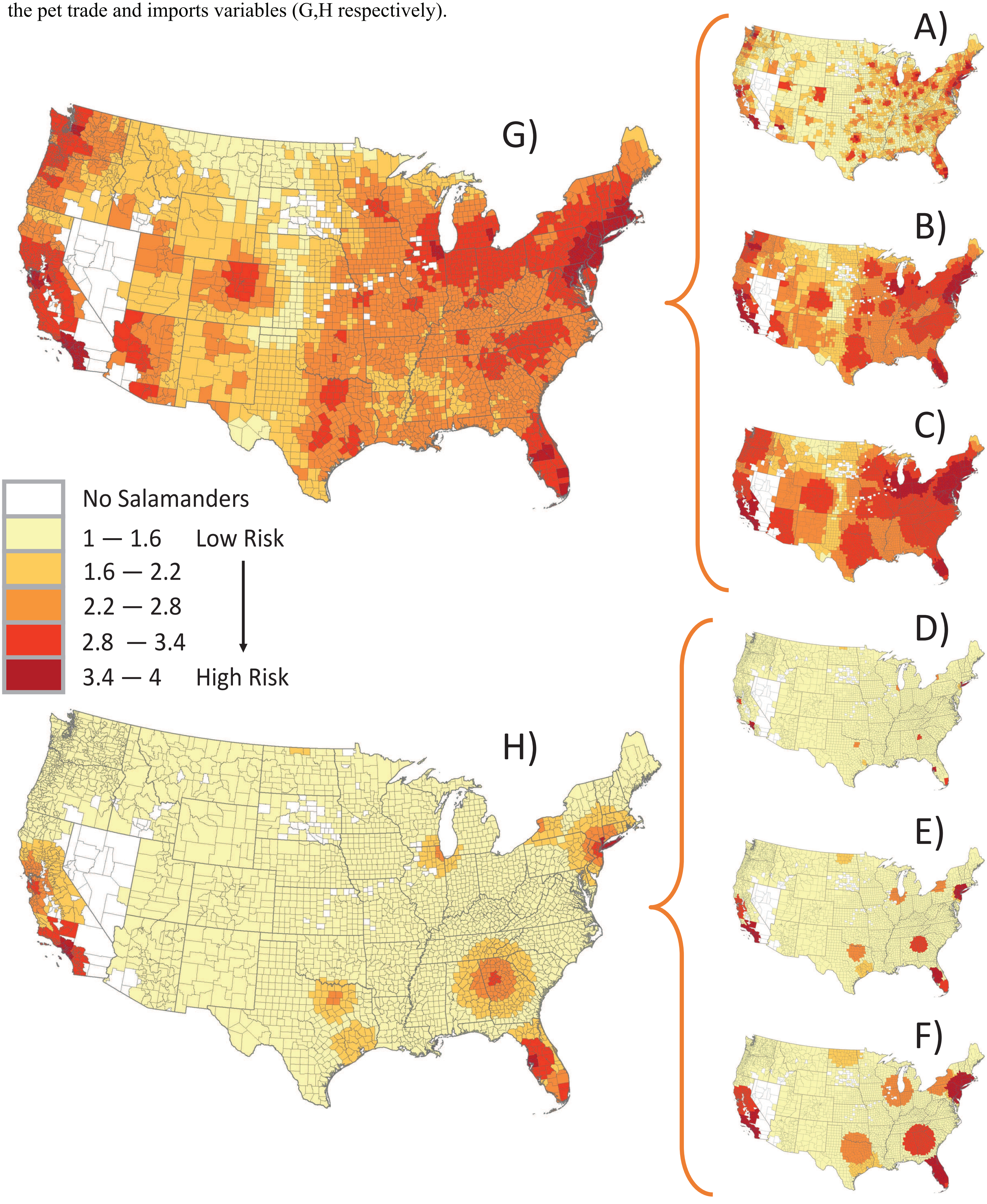

Figure S2: Heat maps of the US showing the two components of environmental suitability for Bsal introduction (A), mean temperature of the hottest month (B) and mean annual temperature (C).

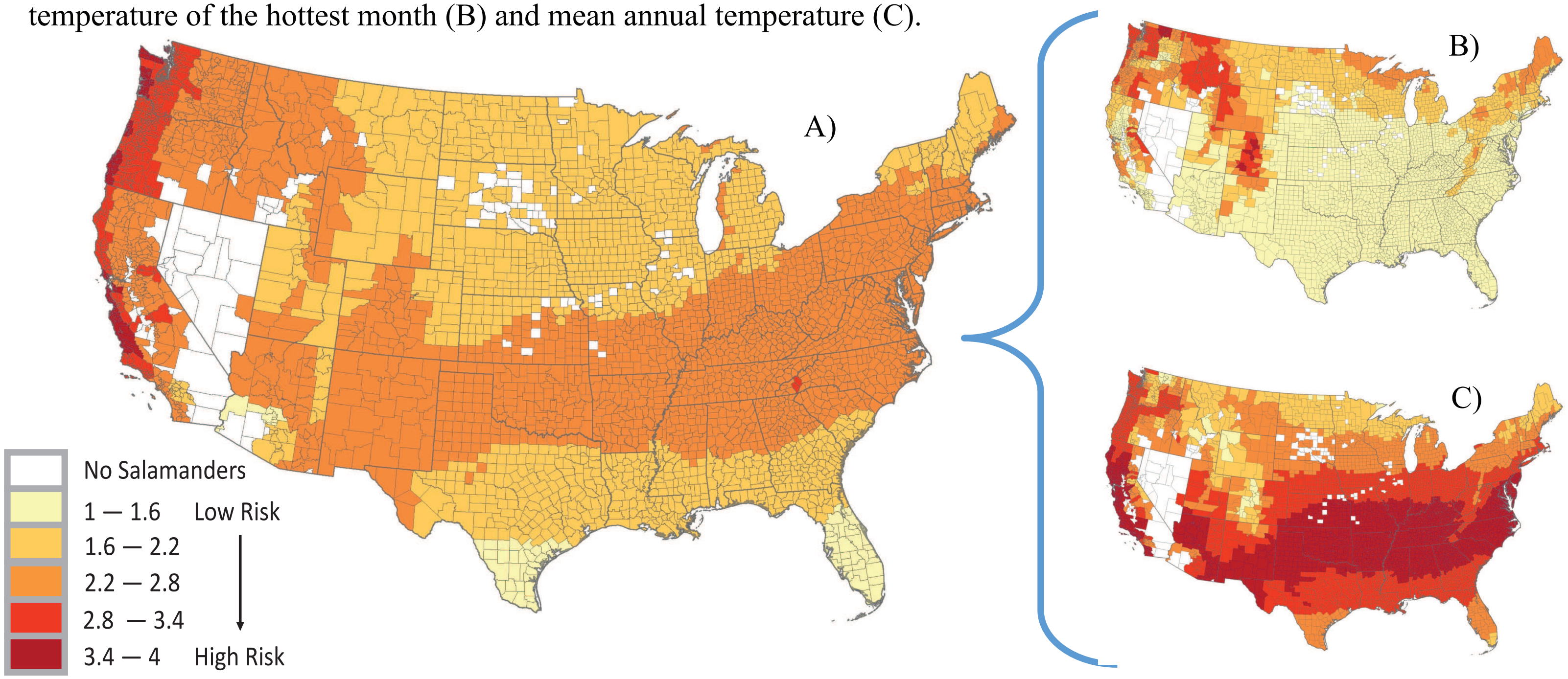

Figure S3: A map of the United States showing the ranges of the two salamander species that had lethal responses to *Bsal* in laboratory exposure trials (Martel et al. 2014).

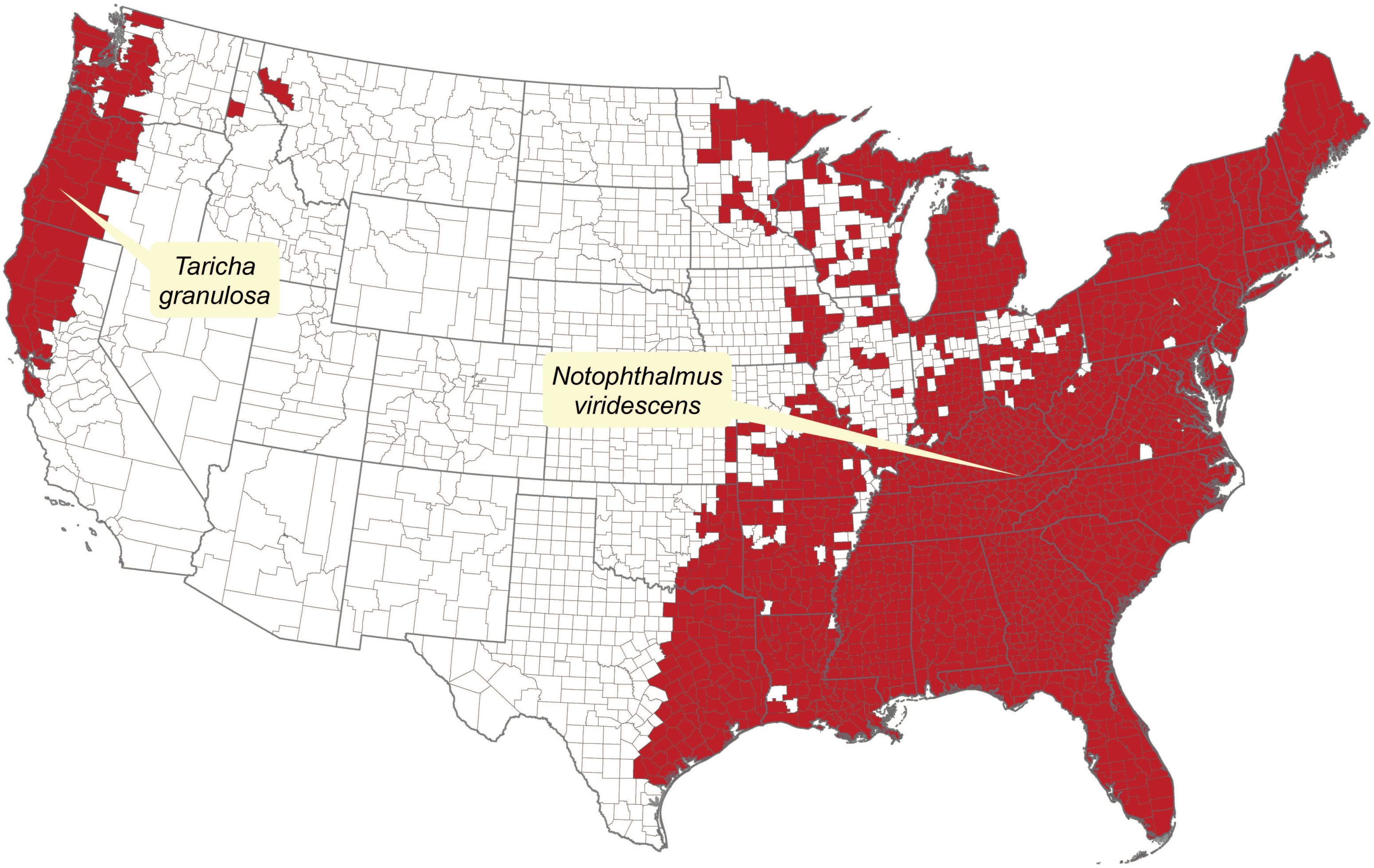

References

Lips, K. R., Diffendorfer, J., Mendelson, J. R. & Sears, M. W. 2008 Riding the wave: reconciling the roles of disease and climate change in amphibian declines. PLoS Biol. 6, e72. (doi:10.1371/journal.pbio.0060072)

Martel, A. et al. 2014 Recent introduction of a chytrid fungus endangers Western palearctic salamanders. Science 346, 630–1. (doi:10.1126/science.1258268)
